# Redetermination of Hg_2_I_2_

**DOI:** 10.1107/S1600536811056339

**Published:** 2012-01-07

**Authors:** Mohammed Kars, Thierry Roisnel, Vincent Dorcet, Allaoua Rebbah, Otero-Diáz L. Carlos

**Affiliations:** a Université Houari-Boumedienne, Faculté de Chimie, Laboratoire Sciences des Matériaux, Bp 32 El-Alia Bab-Ezzouar, Algeria; b Centre de Diffractométrie X, Sciences Chimiques de Rennes, UMR 6226 CNRS Université de Rennes 1, Campus de Beaulieu, Avenue du Général Leclerc, France; c Departomento Inorgánica, Facultad C.C. Químicas, Universidad Complutense, 28040 Madrid, Spain

## Abstract

The crystal structure of mercurous iodide, Hg_2_I_2_, has been determined previously from X-ray powder diffraction data [Havighurst (1926 ▶). *J. Am. Chem. Soc.* **48**, 2113–2125]. The results of the current redetermination based on single-crystal X-ray diffraction data provide more precise geometrical data and also anisotropic displacement parameters for the Hg and I atoms, which are both situated on positions with site-symmetry 4*mm*. The structure consists of linear dimers I—Hg—Hg—I extending along the *c* axis with an Hg—Hg distance of 2.5903 (13) Å. The overall coordination sphere of the Hg^+^ atom is a considerably distorted octa­hedron. The crystal specimen under investigation was twinned by non-merohedry with a refined twin domain fraction of 0.853 (14):0.147 (14).

## Related literature

The structures of the mercurous halides Hg_2_ *X*_2_ (*X* = Cl, Br, I) were originally determined from X-ray powder data by Havighurst (1926 ▶). Studies based on single crystals for *X* = F, Cl, Br were reported by Dorm (1970 ▶) and for *X* = F also by Grdenić & Djordjević (1956 ▶) and Schrötter & Müller (1992 ▶). For the physical properties of mercurous halides, see: Zadokhin & Solodovnik (2004 ▶); Markov *et al.* (2007 ▶, 2010 ▶); Taylor *et al.* (2011 ▶). For theoretical studies of the structures of mercurous halides, see: Liao & Zhang (1995 ▶); Liao & Schwarz (1997 ▶).

## Experimental

### 

#### Crystal data

Hg_2_I_2_
*M* *_r_* = 654.98
Tetragonal, 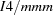

*a* = 4.8974 (9) Å
*c* = 11.649 (2) Å
*V* = 279.40 (9) Å^3^
*Z* = 2
Mo *K*α radiation
μ = 65.76 mm^−1^
*T* = 150 K
0.10 × 0.06 × 0.04 mm

#### Data collection

Bruker APEXII CCD diffractometer
Absorption correction: multi-scan (*TWINABS*; Bruker, 2006 ▶) *T*_min_ = 0.024, *T*_max_ = 0.072
448 measured reflections
253 independent reflections
164 reflections with *I* > 3σ(*I*)
*R*_int_ = 0.056

#### Refinement

*R*[*F* ^2^ > 3σ(*F* ^2^)] = 0.039
*wR*(*F* ^2^) = 0.044
*S* = 1.47
253 reflections
9 parameters
Δρ_max_ = 4.99 e Å^−3^
Δρ_min_ = −4.73 e Å^−3^

### 

Data collection: *APEX2* (Bruker, 2006 ▶); cell refinement: *SAINT* (Bruker, 2006 ▶); data reduction: *SAINT* and *CELL_NOW* (Bruker, 2006 ▶); program(s) used to solve structure: *SIR97* (Altomare *et al.*, 1999 ▶); program(s) used to refine structure: *JANA2006* (Petříček *et al.*, 2006 ▶); molecular graphics: *DIAMOND* (Brandenburg & Putz, 2005 ▶); software used to prepare material for publication: *JANA2006*.

## Supplementary Material

Crystal structure: contains datablock(s) global, I. DOI: 10.1107/S1600536811056339/wm2566sup1.cif

Structure factors: contains datablock(s) I. DOI: 10.1107/S1600536811056339/wm2566Isup2.hkl

Additional supplementary materials:  crystallographic information; 3D view; checkCIF report

## Figures and Tables

**Table 1:** Selected bond lengths (Å)

Hg1—I1	2.7266 (17)
Hg1—I1^i^	3.5000 (9)

Symmetry code: (i) 
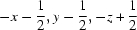
.

**Fig. 1.**
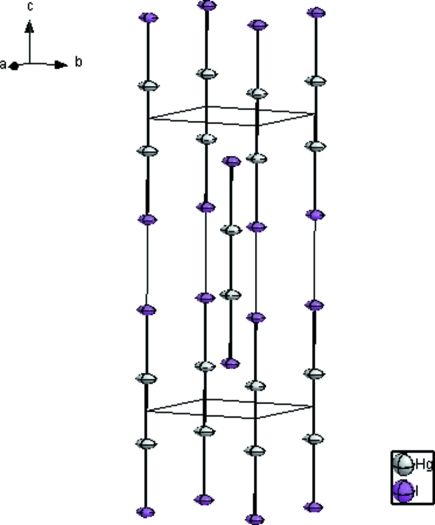
Structure de Hg2I2 montrant les chaînes linéaire I—Hg—Hg—I le long de l'axe c, avec un déplacement des ellipsoīdes à 80% de probabilité.

**Fig. 2.**
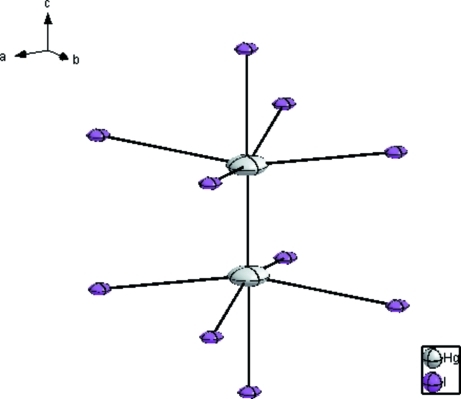
Environnement octaédrique distordu des atomes de Hg, avec un déplacement des ellipsoīdes à 80% de probabilité.

## supplementary crystallographic information

### Comment

Les cristaux d'halogénures de mercure(I) de formulation Hg_2_*X*_2_ (*X* = F, Cl, Br et I) sont connus pour leurs propriétés physiques anisotropes (acousto-optiques, biréfringences *etc*). Ces dernières années, ces composés ont fait l'objet de nombreux travaux, essentiellement pour leurs propriétés dynamiques (Zadokhin & Solodovnik, 2004), ferroélastiques (Markov *et al.* 2007, 2010) et résonnances magnétiques nucléaires (NMR) (Taylor *et al.*, 2011). La présence de liaisons Hg—Hg, qui est l'une des caractéristiques de ce type de composé a suscité plusieurs études quant à la nature et aux propriétés de ces liaisons (Liao & Zhang, 1995; Liao & Schwarz, 1997). La structure de ces halogènures a été déterminée au préalable par diffraction des rayons *X* sur poudre (Havighurst, 1926), puis sur monocristal pour Hg_2_*X*_2_ (*X*: F, Cl et Br) par Dorm (1970) et pour Hg_2_F_2_ par Schrötter & Müller (1992). La structure de Hg_2_I_2_ est analogue à celle des autres halogènures, elle est caractérisée par des chaînes linéaires I—Hg—Hg—I le long de l'axe *c* (Fig. 1). A la différence des autres halogènures formés par les ions Hg_2_^+2^ et *X*^-^, l'iodure de mercure(I) Hg_2_I_2_ quant à lui est caractérisé par des dimères 'HgI' (Grdenić & Djordjević, 1956).

La présente redétermination par diffraction des rayons *X* sur monocristal, fournit plus de précisions sur les distances interatomiques, ainsi que sur les paramètres d'agitation thermiques (ADP's). Les liaisons Hg—Hg [2.5903 (13) Å] et Hg—I [2.7266 (17) Å] sont différentes de celles observées par Havighurst (1926) [respectivement 2.694 et 2.682 Å]. Quatres longues distances Hg—I de 3.5000 (9) Å sont aussi observées, donnant aux atomes de Hg un environnement octadérique distordu (Fig. 2). Les résultats obtenus sur monocristal montrent une tendance des différentes liaisons à varier selon l'électronégativité de l'halogène (Havighurst, 1926). En effet, la liaison Hg—halogéne augmente du fluor à l'iode d'environ 0.58 et 0.78 Å respectivement pour la courte et la longue distance: Hg—F [2.14 (2) et 2.715 (5) Å]; Hg—Cl [2.43 (4) et 3.209 (6) Å]; Hg—Br [2.71 (2) et 3.32 (1) Å] et Hg—I [2.7266 (17) et 3.5000 (9) Å]; quant à liaison Hg—Hg, elle augmente seulement de 0.08 Å: F [2.507 (1) Å]; Cl [2.526 (6) Å]; Br [2.49 (1) Å] et I [2.5903 (13) Å]. Ceci contredit en partie les conclusions de Liao & Zhang, (1995) and Liao & Schwarz (1997), selon lesquelles la liaison Hg—Hg est indépendante de l'électronégativité de l'halogéne. Toutefois une redétermination structurale de Hg_2_Br_2_ semble nécessaire pour confirmer cette tendance. Enfin, il faut remarquer que comme pour les autres halogénures de mercures(I), l'agitation thermique (ADP's) autour du mercure *U*_11_(Hg) est supérieure à *U*_33_(Hg), alors qu'autour de l'iode elle est comparable à celles des autres halogènes.

### Experimental

Les monocristaux de Hg_2_I_2_ ont été obtenus lors des essais de synthèse du clathrate I_8_Hg_10_Ge_36_, à partir d'un mélange d'éléments purs et en présence de HgO et WO_3_ comme agents précurseurs. Le mélange broyé puis scellé dans un tube en quartz, est porté à une température de 1073 K pendant environ 10 jours. Des cristaux de la phase α-Ge ont été aussi identifiés.

### Refinement

La structure a été déterminée par isotypie aux halogénures de mercure(I) dans le groupe d'espace I4/*mmm*. Le cristal étudié correspond à une macle non-mériédrique, contitué de deux individus mis en évidence par le programme *CELL_NOW* (Bruker, 2006) avec une matrice de macle de [0.137 0.983 0.053, 0.984 -0.149 0.038, 0.250 0.262 -0.988]. La structure a été affinée avec JANA2006 en utilisant un fichier de type *HKL5* crée par le programme *TWINABS* (Bruker, 2006) et contenant la contribution de ces deux individus. En fin d'affinement, la fraction en volume des composants est de: 0.853 (14): 0.147 (14), et la carte de densité électronique est de: ρ_max_ = 4.99 e Å^-3^ (localisée à 0.88 Å de I) et ρ_min_ = -4.73 e Å^-3^ (localisée à 1.99 Å de Hg).

### Crystal data

**Table:**

Hg_2_I_2_	*D*_x_ = 7.783 Mg m^−^^3^
*M**_r_* = 654.98	Mo *K*α radiation, λ = 0.71073 Å
Tetragonal, *I*4/*m**m**m*	Cell parameters from 274 reflections
Hall symbol: -I 4 2	θ = 4.5–32.9°
*a* = 4.8974 (9) Å	µ = 65.76 mm^−^^1^
*c* = 11.649 (2) Å	*T* = 150 K
*V* = 279.40 (9) Å^3^	Prism, black
*Z* = 2	0.10 × 0.06 × 0.04 mm
*F*(000) = 532	

### Data collection

**Table:**

Bruker APEXII CCD diffractometer	253 independent reflections
Radiation source: X-ray tube	164 reflections with *I* > 3σ(*I*)
graphite	*R*_int_ = 0.056
ω and φm scans	θ_max_ = 39.6°, θ_min_ = 3.5°
Absorption correction: multi-scan (*TWINABS*; Bruker, 2006)	*h* = −5→6
*T*_min_ = 0.024, *T*_max_ = 0.072	*k* = 0→8
448 measured reflections	*l* = 0→20

### Refinement

**Table:**

Refinement on *F*	0 constraints
*R*[*F*^2^ > 2σ(*F*^2^)] = 0.039	Weighting scheme based on measured s.u.'s *w* = 1/(σ^2^(*F*) + 0.0001*F*^2^)
*wR*(*F*^2^) = 0.044	(Δ/σ)_max_ = 0.0003
*S* = 1.47	Δρ_max_ = 4.99 e Å^−^^3^
253 reflections	Δρ_min_ = −4.73 e Å^−^^3^
9 parameters	Extinction correction: B-C type 1 Gaussian isotropic (Becker & Coppens, 1974)
0 restraints	Extinction coefficient: 27 (7)

### Special details

**Table:**

Refinement. The refinement was carried out against all reflections. The conventional *R*-factor is always based on *F*. The goodness of fit as well as the weighted *R*-factor are based on *F* and *F*^2^ for refinement carried out on *F* and *F*^2^, respectively. The threshold expression is used only for calculating *R*-factors *etc*. and it is not relevant to the choice of reflections for refinement. The crystal studied was twinned by non-merohedry with a refined twin domain fraction of 0.853 (14): 0.147 (14). The program used for refinement, *Jana2006*, uses the weighting scheme based on the experimental expectations, see _refine_ls_weighting_details, that does not force *S* to be one. Therefore the values of *S* are usually larger than the ones from the *SHELX* program.

### Fractional atomic coordinates and isotropic or equivalent isotropic displacement parameters (Å^2^)

**Table:**

	*x*	*y*	*z*	*U*_iso_*/*U*_eq_	
Hg1	0	0	0.11118 (6)	0.0263 (2)	
I1	0	0	0.34524 (10)	0.0207 (3)	

### Atomic displacement parameters (Å^2^)

**Table:**

	*U*^11^	*U*^22^	*U*^33^	*U*^12^	*U*^13^	*U*^23^
Hg1	0.0356 (4)	0.0356 (4)	0.0078 (3)	0	0	0
I1	0.0271 (6)	0.0271 (6)	0.0079 (5)	0	0	0

### Geometric parameters (Å, °)

**Table:**

Hg1—Hg1^i^	2.5903 (13)	Hg1—I1^iii^	3.5000 (9)
Hg1—I1	2.7266 (17)	Hg1—I1^iv^	3.5000 (9)
Hg1—I1^ii^	3.5000 (9)	Hg1—I1^v^	3.5000 (9)
			
Hg1^i^—Hg1—I1	180.0 (5)	I1^iv^—Hg1—I1^v^	88.795 (15)
Hg1^i^—Hg1—I1^ii^	98.34 (2)	Hg1—I1—Hg1^ii^	98.34 (2)
Hg1^i^—Hg1—I1^iii^	98.34 (2)	Hg1—I1—Hg1^iii^	98.34 (2)
Hg1^i^—Hg1—I1^iv^	98.34 (2)	Hg1—I1—Hg1^iv^	98.34 (2)
Hg1^i^—Hg1—I1^v^	98.34 (2)	Hg1—I1—Hg1^v^	98.34 (2)
I1—Hg1—I1^ii^	81.66 (2)	Hg1—I1—I1^vi^	180.0 (5)
I1—Hg1—I1^iii^	81.66 (2)	Hg1^ii^—I1—Hg1^iii^	88.795 (16)
I1—Hg1—I1^iv^	81.66 (2)	Hg1^ii^—I1—Hg1^iv^	88.795 (16)
I1—Hg1—I1^v^	81.66 (2)	Hg1^ii^—I1—Hg1^v^	163.32 (4)
I1^ii^—Hg1—I1^iii^	88.795 (15)	Hg1^ii^—I1—I1^vi^	81.66 (2)
I1^ii^—Hg1—I1^iv^	88.795 (15)	Hg1^iii^—I1—Hg1^iv^	163.32 (4)
I1^ii^—Hg1—I1^v^	163.32 (4)	Hg1^iii^—I1—Hg1^v^	88.795 (16)
I1^iii^—Hg1—I1^iv^	163.32 (4)	Hg1^iii^—I1—I1^vi^	81.66 (2)
I1^iii^—Hg1—I1^v^	88.795 (15)	Hg1^iv^—I1—Hg1^v^	88.795 (16)

Symmetry codes: (i) −*x*, *y*, −*z*; (ii) −*x*−1/2, *y*−1/2, −*z*+1/2; (iii) −*x*−1/2, *y*+1/2, −*z*+1/2; (iv) −*x*+1/2, *y*−1/2, −*z*+1/2; (v) −*x*+1/2, *y*+1/2, −*z*+1/2; (vi) −*x*, *y*, −*z*+1.
